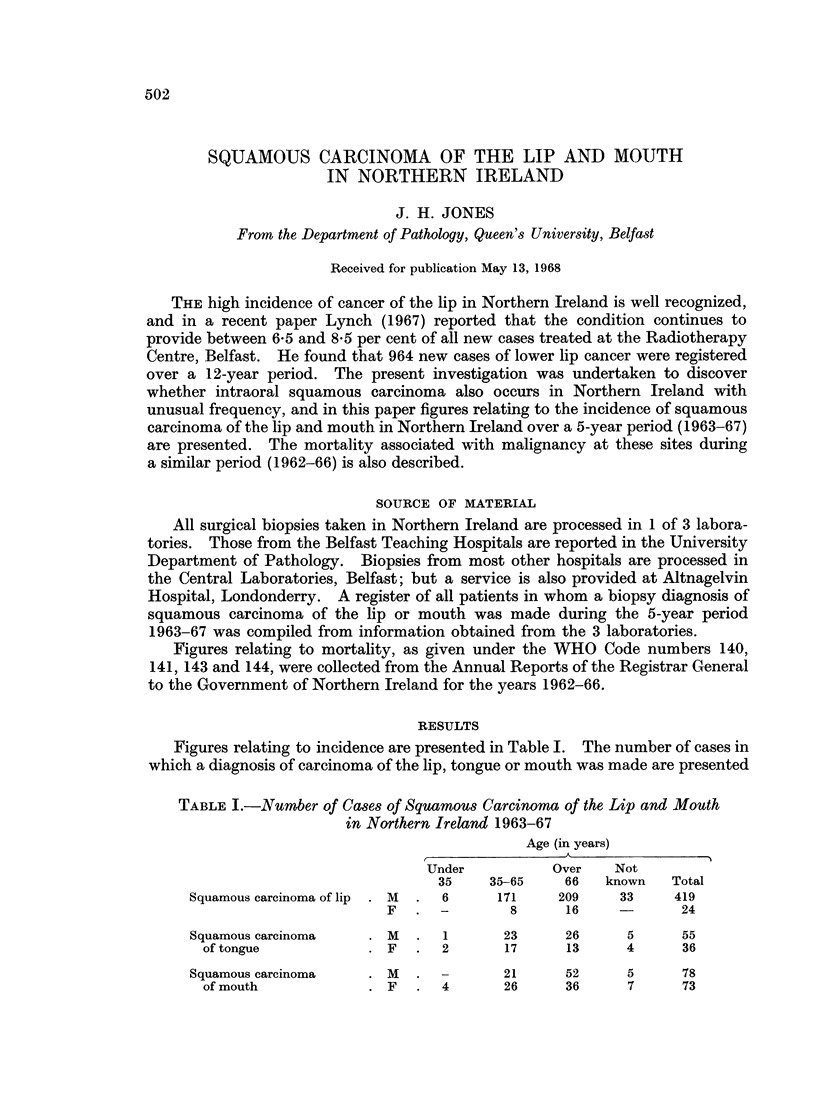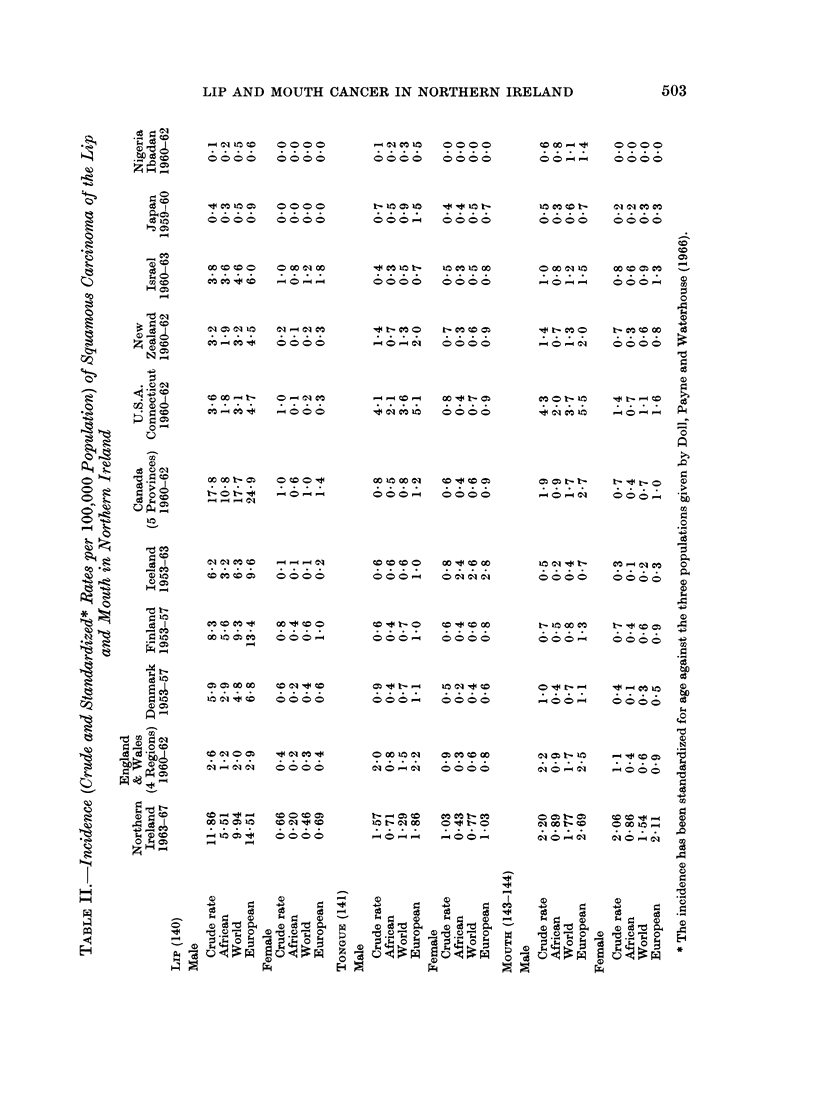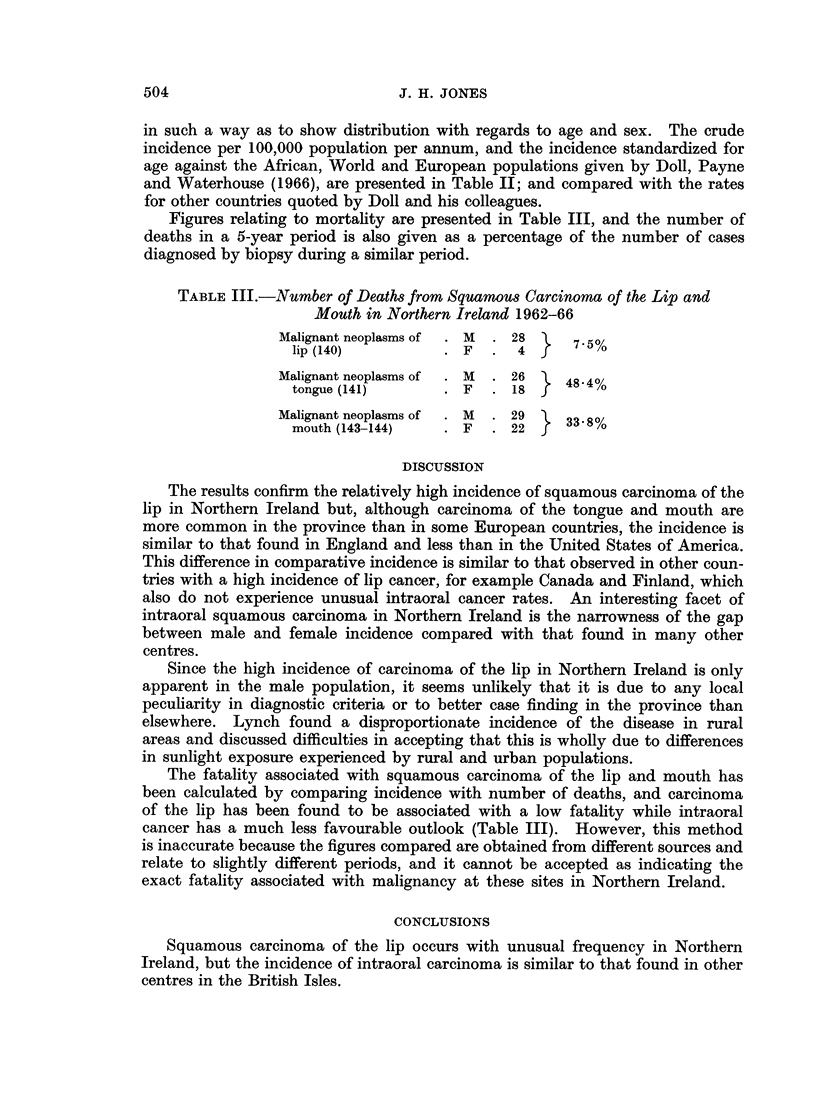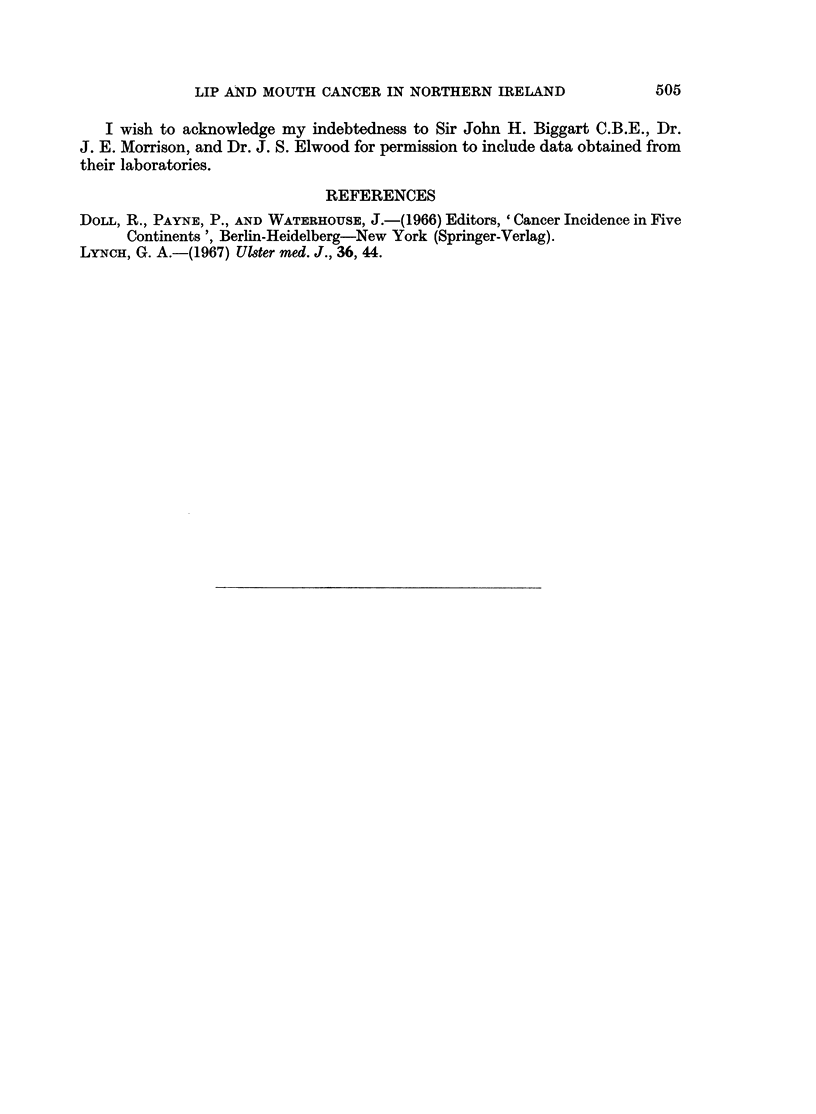# Squamous carcinoma of the lip and mouth in Northern Ireland.

**DOI:** 10.1038/bjc.1968.59

**Published:** 1968-09

**Authors:** J. H. Jones


### SQUAMOUS CARCINOMA OF THE LIP AND MOUTH IN NORTHERN IRELAND

J. H. JONES

*From the Department of Pathology, Queen's University, Belfast*

Received for publication May 13, 1968

THE high incidence of cancer of the lip in Northern Ireland is well recognized, and in a recent paper Lynch (1967) reported that the condition continues to provide between 6·5 and 8·5 per cent of all new cases treated at the Radiotherapy Centre, Belfast. He found that 964 new cases of lower lip cancer were registered over a 12-year period. The present investigation was undertaken to discover whether intraoral squamous carcinoma also occurs in Northern Ireland with unusual frequency, and in this paper figures relating to the incidence of squamous carcinoma of the lip and mouth in Northern Ireland over a 5-year period (1963–67) are presented. The mortality associated with malignancy at these sites during a similar period (1962–66) is also described.

### SOURCE OF MATERIAL

All surgical biopsies taken in Northern Ireland are processed in 1 of 3 laboratories. Those from the Belfast Teaching Hospitals are reported in the University Department of Pathology. Biopsies from most other hospitals are processed in the Central Laboratories, Belfast; but a service is also provided at Altnagelvin Hospital, Londonderry. A register of all patients in whom a biopsy diagnosis of squamous carcinoma of the lip or mouth was made during the 5-year period 1963–67 was compiled from information obtained from the 3 laboratories.

Figures relating to mortality, as given under the WHO Code numbers 140, 141, 143 and 144, were collected from the Annual Reports of the Registrar General to the Government of Northern Ireland for the years 1962–66.

### RESULTS

Figures relating to incidence are presented in Table I. The number of cases in which a diagnosis of carcinoma of the lip, tongue or mouth was made are presented

TABLE I.—*Number of Cases of Squamous Carcinoma of the Lip and Mouth in Northern Ireland* 1963–67

| | | Age (in years) | | | | |
|---|---|---|---|---|---|---|
| | | Under 35 | 35–65 | Over 66 | Not known | Total |
| Squamous carcinoma of lip | M | 6 | 171 | 209 | 33 | 419 |
| | F | – | 8 | 16 | — | 24 |
| Squamous carcinoma of tongue | M | 1 | 23 | 26 | 5 | 55 |
| | F | 2 | 17 | 13 | 4 | 36 |
| Squamous carcinoma of mouth | M | – | 21 | 52 | 5 | 78 |
| | F | 4 | 26 | 36 | 7 | 73 |

in such a way as to show distribution with regards to age and sex. The crude incidence per 100,000 population per annum, and the incidence standardized for age against the African, World and European populations given by Doll, Payne and Waterhouse (1966), are presented in Table II; and compared with the rates for other countries quoted by Doll and his colleagues.

Figures relating to mortality are presented in Table III, and the number of deaths in a 5-year period is also given as a percentage of the number of cases diagnosed by biopsy during a similar period.

TABLE III.—*Number of Deaths from Squamous Carcinoma of the Lip and Mouth in Northern Ireland* 1962–66

| | | | |
|---|---|---|---|
| Malignant neoplasms of lip (140) | M | 28 | 7·5% |
| | F | 4 | |
| Malignant neoplasms of tongue (141) | M | 26 | 48·4% |
| | F | 18 | |
| Malignant neoplasms of mouth (143–144) | M | 29 | 33·8% |
| | F | 22 | |

### DISCUSSION

The results confirm the relatively high incidence of squamous carcinoma of the lip in Northern Ireland but, although carcinoma of the tongue and mouth are more common in the province than in some European countries, the incidence is similar to that found in England and less than in the United States of America. This difference in comparative incidence is similar to that observed in other countries with a high incidence of lip cancer, for example Canada and Finland, which also do not experience unusual intraoral cancer rates. An interesting facet of intraoral squamous carcinoma in Northern Ireland is the narrowness of the gap between male and female incidence compared with that found in many other centres.

Since the high incidence of carcinoma of the lip in Northern Ireland is only apparent in the male population, it seems unlikely that it is due to any local peculiarity in diagnostic criteria or to better case finding in the province than elsewhere. Lynch found a disproportionate incidence of the disease in rural areas and discussed difficulties in accepting that this is wholly due to differences in sunlight exposure experienced by rural and urban populations.

The fatality associated with squamous carcinoma of the lip and mouth has been calculated by comparing incidence with number of deaths, and carcinoma of the lip has been found to be associated with a low fatality while intraoral cancer has a much less favourable outlook (Table III). However, this method is inaccurate because the figures compared are obtained from different sources and relate to slightly different periods, and it cannot be accepted as indicating the exact fatality associated with malignancy at these sites in Northern Ireland.

### CONCLUSIONS

Squamous carcinoma of the lip occurs with unusual frequency in Northern Ireland, but the incidence of intraoral carcinoma is similar to that found in other centres in the British Isles.

I wish to acknowledge my indebtedness to Sir John H. Biggart C.B.E., Dr. J. E. Morrison, and Dr. J. S. Elwood for permission to include data obtained from their laboratories.

### REFERENCES

Doll, R., Payne, P., and Waterhouse, J.—(1966) Editors, 'Cancer Incidence in Five Continents', Berlin-Heidelberg—New York (Springer-Verlag).

Lynch, G. A.—(1967) *Ulster med. J.*, **36**, 44.